# Developing the Chinese version of the new 5-level EQ-5D descriptive system: the response scaling approach

**DOI:** 10.1007/s11136-012-0200-0

**Published:** 2012-05-24

**Authors:** Nan Luo, Minghui Li, Gordon G. Liu, Andrew Lloyd, Frank de Charro, Michael Herdman

**Affiliations:** 1 Saw Swee Hock School of Public Health, National University of Singapore, 16 Medical Drive, Block MD3, Singapore, 117597 Singapore; 2 Department of Pharmaceutical Health Services Research, University of Maryland School of Pharmacy, Baltimore, MD USA; 3 Guanghua School of Management, Peking University, Beijing, China; 4 Oxford Outcomes, Oxford, United Kingdom; 5 EuroQol Group Executive Office, Rotterdam, The Netherlands; 6 Insight Consulting and Research, Mataró, Spain

**Keywords:** Cultural adaptation, EQ-5D, Health-status instrument, Response scaling

## Abstract

**Purpose:**

To develop a Chinese version of the new, 5-level EQ-5D descriptive system (EQ-5D-5L) from the existing EQ-5D-3L by identifying Chinese label wording suitable for constructing EQ-5D-5L’s 5-point response scales.

**Methods:**

In face-to-face interviews, perceived severity of selected Chinese labels when they were used to describe EQ-5D health problems was measured from 50 native Chinese speakers using a 0 (no problems) to 100 (the worst problems) visual analog scale. Selection of label wording was based on the severity scores and semantic similarity with label wording used in the existing English and Spanish EQ-5D-5L.

**Results:**

The severity scores supported the use of Chinese wording of ‘only a little’ (range of median: 12.5–17), ‘moderate’ (range of median: 50–53), and ‘severe’ (range of median: 82.5–90) as the descriptors for the intermediate functional levels of the five EQ-5D dimensions and the label wording of ‘very severe’ (median: 90) to describe the worst level of pain/discomfort and anxiety/depression.

**Conclusions:**

The Chinese version of the EQ-5D-5L comprises descriptors with similar interpretations as those used its English and Spanish counterparts. The response scaling exercise is a useful method for cross-cultural adaptation of health-status instruments.

## Background

The EQ-5D is an instrument for valuing health in terms of the dimensions of mobility (MO), self-care (SC), usual activities (UA), pain/discomfort (PD), and anxiety/depression (AD). The EQ-5D-3L is the first version of this preference-based instrument. It comprises five items, one for each of the above dimensions, and each item contains 3 descriptors (corresponding to no, some, and extreme problems) that allow respondents to self-rate their health. Although extensively used in health-related research, the EQ-5D-3L has demonstrated some ceiling effects [1–7] and some degree of measurement insensitivity [5, 8–10]. In order to try to improve the instrument’s discriminative capacity and sensitivity to change, the EuroQol Group recently developed a new, 5-level version of the questionnaire (EQ-5D-5L); the new version was developed in parallel in English (UK) and Spanish (Spain) [11]. The EQ-5D-5L descriptive system incorporates the same health dimensions as the original 3L version, but uses a 5-point response scale (i.e., no, slight, moderate, severe, and extreme problems) in place of the earlier 3 levels of severity [12, 13].

The present study aimed to develop a Chinese version of the EQ-5D-5L. Instead of simply translating it from English into Chinese, we quantitatively assessed possible wording for Chinese response options using the response scaling method to ensure similar interpretations of the EQ-5D-5L response scales between the Chinese and the existing versions.

## Methods

### Generation of candidate labels

The core task was to determine the Chinese label wording for the three intermediate levels (i.e., ‘slight/moderate/severe’) of the 5-point response scale for the EQ-5D functional dimensions (i.e., MO, SC, and UA) and the wording for four of the five levels (i.e., ‘slight/moderate/severe/extreme’) in the two EQ-5D symptom dimensions (i.e., PD and AD). The anchors of the EQ-5D-5L response scales for the functional dimensions and the upper anchors for the symptom dimensions were from the EQ-5D-3L except for the lower anchor for MO (‘I am unable to walk about’) which is worded as ‘I am confined to bed’ in EQ-5D-3L.

We collected a set of potential Chinese response labels for the EQ-5D-5L using two approaches. First, we translated into Chinese all of the labels tested in the development of the (UK) English EQ-5D-5L using a forward and backward translation procedure. A total of 11 and 9 labels describing various levels of severity were generated for the three functional dimensions and the two symptom dimensions, respectively, during development of the English EQ-5D-5L [11]. Second, we reviewed existing Chinese health-status questionnaires and searched Chinese dictionaries and thesauri for potentially useful labels.

### Response scaling exercise

Once a set of potential labels had been identified for each dimension, we used the response scaling method to elicit the opinion of native Chinese speakers regarding the level of severity represented by each label. Native Chinese speakers were recruited from a large shopping mall located in downtown Beijing (China) during a weekend. A quota sampling method was used to recruit equal numbers of men and women aged between 18 and 70 years. Consenting adults were interviewed in a conference room in the shopping mall to complete the response scaling exercise which is a method for quantifying the magnitude of attribute that a response scale’s label wording represents from respondents [14, 15]. The standard interview protocol developed by the EuroQol Group was used in this study [16]. It is briefly described below.

In face-to-face interviews, each participant was asked first to rank the labels according to the severity they represent and then to assign each label a score from 0 (no problems) to 100 (the worst problems) using a visual analog scale (VAS). Labels were rated by health dimension. To facilitate the ranking and rating exercise, each label was printed on a separate card and a 40 cm vertical, hash-marked VAS was placed in front of each respondent during the interview. Labels were presented to the participants in random order to minimize possible ordering effects.

Professional interviewers were trained by the investigators and conducted at least one trial interview before the main study. Each participant was given a thank-you gift on completion of the interview.

### Selection of response labels

The perceived severity of each label was estimated by calculating the median (inter-quartile range) rating score. The selection criteria for the labels for the three intermediate levels were pre-defined as follows: (1) similar perceived severity as that of corresponding labels in the English and Spanish EQ-5D-5L (slight: 15–20; moderate: 40–45; and severe: 75–80) [11]; (2) colloquialism; (3) semantic similarity to their English and Spanish counterparts; and (4) one set of labels applicable to all five dimensions. The criteria were defined so as to achieve comparable measurement scales between the Chinese and the English/Spanish EQ-5D-5L.

## Results

Eight and nine labels were derived from translation of English labels for functional and symptom dimensions, respectively. The English label of ‘moderate problems’ for describing functional levels in the EQ-5D-5L was translated into ‘moderate difficulty’ as the literal translation of ‘moderate problems’ into Chinese was unnatural. For the purpose of consistency, we substituted the phrase ‘problems’ with ‘difficulty’ whenever it appears in a label. Seven and three additional labels were generated for the functional and symptom dimensions, respectively, from existing Chinese literature. Hence, a total of 15 and 12 labels were tested for each functional and symptom dimension, respectively.

Of 51 consenting participants, 50 successfully completed the response scaling exercise; one participant quit half way because of urgent personal issues. The sample characteristics are shown in Table 1. On average, the exercise took 40 min (range: 21–62 min). Ninety-eight of the participants were rated by interviewers as having no (70 %) or some difficulty (28 %) understanding the response scaling exercise; only 1 participant appeared to experience great difficulty.

**Table 1:** Participants’ characteristics

Variable	Level	*n*	%
Sex	Male	25	50
	Female	25	50
Age (year)	18–20	10	20
	21–30	12	24
	31–40	4	8
	41–50	8	16
	51–60	8	16
	61–70	8	16
Education attainment	University/college	14	28
	High school	20	40
	Secondary school	16	32
Employment status	Employee/student	30	60
	Retiree	16	32
	Homemaker/other	4	8

**Fig. 1:**
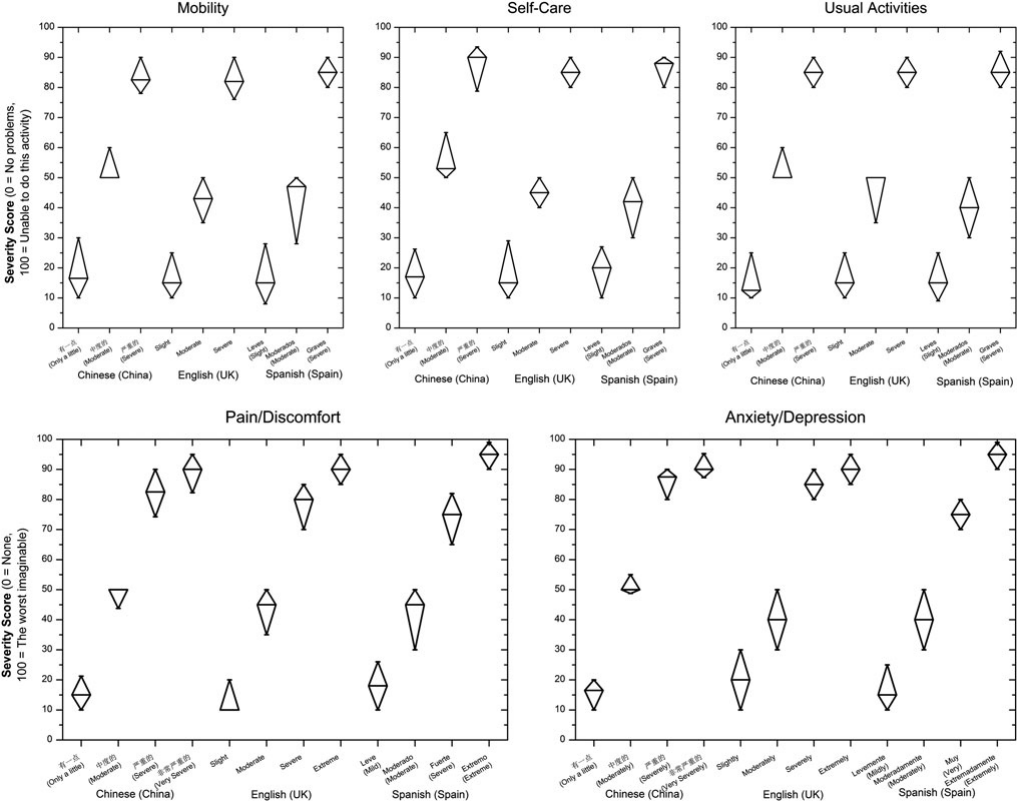
Median (inter-quartile range) ratings of label wording of the Chinese, English, and Spanish versions of the EQ-5D-5L descriptive system. *Horizontal bars* inside the diamonds represent medians and tips are 25th and 75th percentiles

The median severity scores and their inter-quartile ranges for all tested labels are displayed in Tables 2 and 3. As can be seen, ‘moderate’ and ‘severe’ were the best labels for the third and forth response options, respectively, for all dimensions. The labels of ‘slight’ and ‘only a little’ had similar median severity scores and both work with ‘moderate’ and ‘severe’. We decided to choose ‘only a little’ because it is a colloquial phrase understood by poorly educated persons. For the PD and AD dimensions, the label of ‘extreme’ had higher median scores than ‘severe’; however, 10 (20 %) and 8 (16 %) participants assigned ‘severe’ a higher (worse) score than ‘extreme’ when rating these labels for the PD and AD dimensions, respectively. In contrast, 6 (12 %) and 3 (6 %) participants rated ‘severe’ as worse than ‘very severe’ for the PD and AD dimensions, respectively. Given this result and that ‘very severe’ and ‘extreme’ had similar severity scores (Table 3), we decided to choose ‘very severe’ as the label for the worst level of PD and AD. The selected labels were similar to their counterparts in the English and Spanish EQ-5D-5L in median severity scores (Fig. 1). The wording of the recommended Chinese EQ-5D-5L is displayed in the Appendix.

**Table 2:** Median (inter-quartile range) severity scores for labels for functional dimensions of EQ-5D-5L

Label wording (back translation)	Dimension		
	Mobility	Self-care	Usual activities
轻微的困难 (Slight difficulty)	17.5 (10–32)	15 (10–30)	17.5 (9.25–30)
较小的困难 (Minor difficulty)	20 (15–30)	20 (10–30)	20 (15–30.5)
有一点困难 (Only a little difficulty)	16.5 (10–30)	17 (10–26.25)	12.5 (10–25)
很小的困难 (Very little difficulty)	20 (10–26.5)	16.5 (10–30)	11 (10–30)
轻度的困难 (Mild difficulty)	30 (15–40)	30 (20–48.5)	20 (12.25–30)
有些困难 (Some difficulty)	30 (20–50)	39 (25–50)	30 (19.5–40)
中度的困难 (Moderate difficulty)	50 (50–60)	53 (50–65)	50 (50–60)
较大的困难 (Major difficulty)	70 (60–80)	75 (60–80)	70 (60–85)
相当困难 (Quite difficult)	80 (70–80)	75.5 (66–90)	80 (63.75–90)
非常困难 (Very difficult)	80 (74.5–90)	80 (70–90)	85 (73.75–90)
很多困难 (A lot of difficulty)	70 (50–76.25)	70 (60–80)	70 (60–80)
严重的困难 (Severe difficulty)	82.5 (78–90)	90 (78.75–93.5)	85 (80–90)
极度的困难 (Extreme difficulty)	90 (80–95)	90 (83.75–95.25)	90 (80–95)
很大的困难 (Great difficulty)	77.5 (70–85)	80 (68.75–85)	80 (70–90)
极大的困难 (Extremely great difficulty)	90 (80–95)	90 (80–95)	90 (80–95)

**Table 3:** Median (inter-quartile range) severity scores for labels for symptom dimensions of EQ-5D-5L

Label wording (back translation)	Dimension	
	Pain/discomfort	Anxiety/depression
有一点 (Only a little)	15 (10–21.25)	16.5 (10–20)
轻微的 (Slight)	20 (10–25)	20 (10–30)
轻度的 (Mild)	20 (15–30)	22.5 (17.5–32.75)
有些 (Some)	22.5 (15–35)	25 (16.5–36.25)
中度的 (Moderate)	50 (43.75–50)	50 (48.75–55)
很多 (A lot of)	64 (60–80)	60 (53.75–71.25)
相当 (Quite)	70 (58.75–80)	70 (50–80)
非常 (Very)	75 (60–85)	70 (60–80)
严重的 (Severe)	82.5 (74.25–90)	87.5 (80–90)
非常严重的 (Very severe)	90 (82.25–95)	90 (87.25–95.25)
极度的 (Extreme)	90 (82.25–95)	90 (85–95)
重度的 (Serious)	85 (72.5–90)	85 (77.25–90.5)

## Discussion

In this study, we developed a Chinese version of the EQ-5D-5L using a standard protocol developed by the EuroQol Group and found that the label wording of response scales in the new and two existing language versions of the instrument have similar interpretations.

The Chinese EQ-5D-5L we developed is semantically different from its English counterpart in the label wording for level-2 problems (‘only a little’ vs. ‘slight’) for all dimensions and level-5 problems (‘very severe’ vs. ‘extreme’) for the dimensions of PD and AD. The Chinese label of ‘only a little’ was selected because it is a widely used phrase in everyday conversation. Use of colloquial language is important as China has an illiterate population of 114 million [17], and the EQ-5D instrument is designed for both self-completion and interviews. The label ‘only a little’ was also preferred to ‘slight’ because there was less variability in scores for the former, as indicated by the inter-quartile ranges for the two labels (Tables  2 and 3). The Chinese label of ‘very severe’ is recommended as it would work better with the label of ‘severe’ to form an ordinal response scale for PD and AD. The Chinese label of ‘extreme’ was perceived by a sizable of respondents as less undesirable than ‘severe’, indicating that it was not well understood. Based on the median (inter-quartile range) scores (Table 3), ‘very severe’ represents a similar level of severity as ‘extreme’ and therefore can be used to substitute ‘extreme’ without affecting the interpretations of the scale.

Our study demonstrated that response scaling is a useful additional exercise for developing new instruments. However, only a few instruments, such as the SF-36 and WHOQOL, used the response scaling method to formally assess response options [14, 15]. Currently recommended procedures [18] emphasize achievement of semantic equivalence between source and target languages through review of forward and back translations. Such qualitative procedures can neither ensure nor assess scaling equivalence [19] or ordinality of the resultant response scales.

Our sample size was relatively small. However, this is consistent with most existing response scaling studies [11, 14, 15]. Also, our study did not include persons in very poor socio-economic status, since all participants were recruited from a shopping mall. Our plan is to further adapt and test the Chinese EQ-5D-5L developed in this study in Chinese populations outside China.

In conclusion, the Chinese version of the EQ-5D-5L comprises descriptors with similar interpretations as those used its English and Spanish counterparts. The response scaling exercise is a useful method for cross-cultural adaptation of health-status instruments.

## Appendix: The Chinese (China) version of the EQ-5D-5L descriptive system

**四处走动**

我四处走动没有困难

我四处走动有一点困难

我四处走动有中度的困难

我四处走动有严重的困难

我无法四处走动

**自我照顾**

我自己洗澡或穿衣没有困难

我自己洗澡或穿衣有一点困难

我自己洗澡或穿衣有中度的困难

我自己洗澡或穿衣有严重的困难

我无法自己洗澡或穿衣

**日常活动**

我进行日常活动没有困难

我进行日常活动有一点困难

我进行日常活动有中度的困难

我进行日常活动有严重的困难

我无法进行日常活动

**疼痛或不舒服**

我没有疼痛或不舒服

我有一点疼痛或不舒服

我有中度的疼痛或不舒服

我有严重的疼痛或不舒服

我有非常严重的疼痛或不舒服

**焦虑或抑郁**

我没有焦虑或抑郁

我觉得有一点焦虑或抑郁

我觉得中度焦虑或抑郁

我觉得严重焦虑或抑郁

我觉得非常严重的焦虑或抑郁

Note: Intended users of the EQ-5D-5L questionnaire are advised to contact the EuroQoL Group (www.euroqol.org) for the official version.
